# A glimpse into the impact of physical activity on linear growth in children and adolescents through activity restrictions accompanying the COVID-19 outbreak

**DOI:** 10.37796/2211-8039.1696

**Published:** 2026-03-01

**Authors:** Yi-Chun Lin, Wen-Ling Liao, Chung-Hsing Wang, Fuu-Jen Tsai

**Affiliations:** a Department of Chinese Medicine, China Medical University Hospital, Taichung, Taiwan; b College of Public Health, China Medical University, Taichung, Taiwan; c Graduate Institute of Integrated Medicine, School of Chinese Medicine, China Medical University, Taichung, Taiwan; d Center for Personalized Medicine, Department of Medical Research, China Medical University Hospital, Taichung, Taiwan; e Department of Medical Genetics and Pediatric Endocrinology, China Medical University Children’s Hospital, Taichung, Taiwan; f School of Medicine, College of Medicine, China Medical University, Taichung, Taiwan; g School of Chinese Medicine, China Medical University, Taichung, Taiwan; h Department of Medical Research, China Medical University Hospital, Taichung, Taiwan

**Keywords:** Adolescents, Children, COVID-19 lockdown, Linear growth, Physical activity

## Abstract

**Background:**

Linear growth in children and adolescents, a key health indicator, is influenced by various factors, including physical activity (PA). The COVID-19 lockdown in Taiwan created a unique opportunity to examine the effect of PA restrictions on growth.

**Methods:**

This retrospective cohort study was conducted at two hospitals in central Taiwan. The study included 72 children and adolescents (26 males, 46 females) who attended a growth clinic. Height and weight measurements were recorded in three stages: pre-lockdown, during lockdown, and post-lockdown. Growth rates were compared across stages using paired t-tests.

**Results:**

The average growth rate was lowest during lockdown (0.0181 cm/day) and highest pre-lockdown (0.0198 cm/day), although the difference was not statistically significant. In males, growth rates significantly increased post-lockdown compared to those during lockdown (p = 0.04), while no significant differences were observed in females across stages.

**Conclusion:**

PA positively influences linear growth in children, with a more pronounced effect in boys. Lockdown restrictions led to reduced PA, particularly impacting boys’ growth rates. These findings emphasize the importance of PA for healthy growth in children, especially under restrictive conditions, and suggest the need to further encourage active lifestyles among children, particularly girls.

## 1. Introduction

Growth, recognized as a key health indicator for children and adolescents, is evaluated through measurements of weight and height gain [1,2]. Linear growth in children is driven by a complex physiological process known as endochondral ossification, which occurs at the growth plates of long bones, enabling bone elongation. This process involves chondrocyte proliferation and hypertrophy, extracellular matrix secretion, and bone remodeling, all governed by intrinsic mechanisms within the growth plate [3,4]. Research indicates that these mechanisms are influenced by local mediators such as hormones and inflammatory cytokines [3]. Genetic factors, nutrition, endocrine function, metabolism, environmental conditions, psychological factors, disease, and physical activity (PA) all regulate the expression of these mediators and, in turn, affect linear growth indirectly [2,5]. However, to date, no consensus has been reached regarding the direct impact of PA on linear growth in children and adolescents [5,6].

PA is defined as any bodily movement involving skeletal muscles that results in energy expenditure. Physical exercise, a subset of PA, is specifically structured, organized, and planned [7]. The intensity of PA is classified based on the body’s physiological response and can range from light (LPA), moderate (MPA), moderate to vigorous (MVPA) and vigorous intensity activity (VPA) [8]. Studies indicate a positive effect of PA on bone health in children and adolescents. Current guidelines recommend that children and adolescents engage in at least 60 min of moderate-to-high intensity PA daily to support bone health [3,9]. Recent research further suggests that children and adolescents who participate in sports – often exceeding these recommendations – experience a positive impact on linear growth [10]. However, a systematic review found no statistically significant difference in linear growth between children aged 7–12 years who engaged in strength or resistance training and those who did not [11]. Research on the relationship between PA and linear growth in children and adolescents faces notable challenges due to the multiple influencing factors and mediators involved in growth, which affect this susceptible population. Ethical considerations also prevent instructing control groups of children to reduce PA levels. Systematic reviews or meta-analyses are complicated by the specificity of study populations—such as athletes, variability in PA intensities, age differences, varied study durations, and the range of confounding factors [2]. These challenges contribute to the lack of consensus regarding the impact of PA on linear growth in children and adolescents.

During the COVID-19 pandemic, state-enforced social distancing measures significantly limited PA among children and adolescents. School closures reduced opportunities for active commuting, recess play, and physical education classes, which are critical avenues for meeting daily PA recommendations [12]. In parallel, the closure of sports centers, playgrounds, and parks further restricted access to outdoor and PA for this population [13]. Recent meta-analyses indicate a 20 % reduction in the total daily PA levels of children and adolescents from baseline, irrespective of pre-pandemic activity levels. Moderate-to-high intensity PA dropped by up to 28 %, with moderate-to-vigorous activity specifically declining by an average of 17 min per day [12]—nearly one-third of the recommended daily duration for this age group—posing potential long-term health risks for children [14,15]. However, this crisis has also created an unprecedented research opportunity. The extensive and uniform nature of these restrictions has inadvertently established a natural experiment, allowing for unique observations on the effects of PA constraints on linear growth in children and adolescents.

To mitigate the spread of Covid-19 pandemic, the Taiwanese government implemented a lockdown across Taiwan from May 19, 2021, to July 27, 2021, closing campuses, sports centers, playgrounds, entertainment venues, parks, squares, and beaches. Due to epidemic prevention policies, PA among Taiwanese children and adolescents has decreased broadly. We took this opportunity to conduct a retrospective observational study to investigate the impact of PA on linear growth of children and adolescents, by collecting height records of children and adolescents, attending the Child Growth and Development Clinic of a medical center and a children’s hospital in central Taiwan, before and after the period of the level 3 alert in 2021.

## 2. Methods

### 2.1. Research design

This retrospective cohort study was conducted at a medical center (China Medical University Hospital) and the children’s hospital (China Medical University Children’s Hospital) in Central Taiwan. The study involved outpatients visiting the Child Growth and Development Clinic for management or treatment of growth-related issues such as short stature, failure to thrive, precocious puberty, simple obesity, growth hormone deficiency, thyroid disorders, Turner syndrome, and other endocrine and metabolic disorders. This study was conducted following the principles outlined in the Declaration of Helsinki and received approval from the Research Ethics Committee of China Medical University and CMUH [CMUH111-REC2-183]. Conducted retrospectively, this study utilized routine data from the CMUH electronic medical record system, with all data accessed anonymously. Consequently, the institutional review board waived the requirement for informed consent.

### 2.2. Study period and study population

**Fig. 1:**
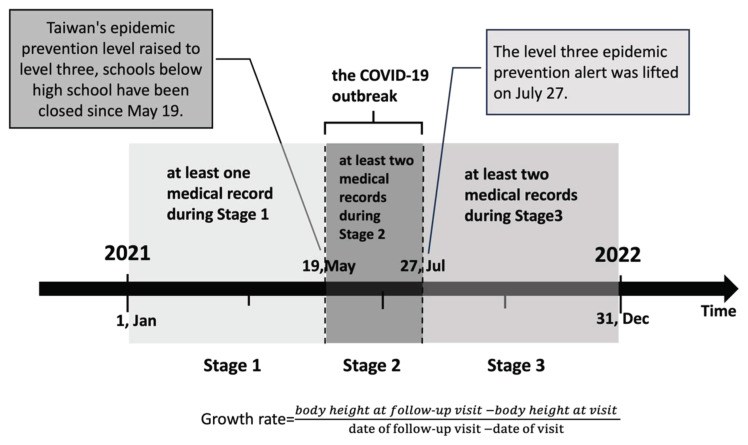
The research design and period for this study spanned from January 1, 2021, to December 31, 2021, and was divided into three distinct stages according to the government’s implementation of lockdown measures due to the COVID-19 outbreak.

Fig. 1 illustrates the study design. The research period for this study spanned from January 1, 2021, to December 31, 2021, and was divided into three distinct stages according to the government’s implementation of lockdown measures due to the COVID-19 outbreak. Stage 1 (S1) encompassed the pre-lockdown period before Alert Level 3 (January 1 to May 18, 2021), Stage 2 (S2) occurred during the lockdown under Alert Level 3 (May 19 to July 27, 2021), and Stage 3 (S3) represented the post-lockdown period after the lifting of Alert Level 3 (July 28 to December 31, 2021). The inclusion criteria for participants were as follows: both male and female patients, with females aged four to fifteen years old and males aged four to seventeen years old, and availability of documented measurements of body weight and height. Participants were required to have at least one medical record during Stage 1, two medical records during Stage 2, and two medical records during Stage 3. If multiple records were available within each stage, the records closest to key dates were selected. The exclusion criteria included patients without documented measurements of weight and height, those with chronic progressive diseases, or newly diagnosed with malignancies, chronic illnesses (such as type 1 diabetes, chronic renal failure, chronic heart disease, or eating disorders) during the study period. Additional exclusion criteria covered patients with mental health conditions, genetic syndromes (e.g., Prader–Willi syndrome, Alström syndrome), endocrine disorders, and those with medication changes or surgeries within the research period. Finally, girls with a bone age exceeding 15 years and boys with a bone age exceeding 17 years, or those at Tanner stage B5P5/G5P5, were excluded from the study.

### 2.3. Anthropometric measurements

Anthropometric measurements were taken for all participants. Body weight was measured to the nearest 0.1 kg using a calibrated balance beam scale, and body height was measured to the nearest 0.1 cm. Body mass index (BMI) was calculated by dividing body weight (in kilograms) by the square of body height (in meters). The growth rate of each case in the three stages was calculated by the following formula.

All measurements were performed by trained healthcare professionals (nurses/physicians) using professionally validated equipment.


$$growth\;rate=\frac{body\;height\;at\;followup\;visit-body\;height\;at\;visit}{date\;of\;followup\;visit-date\;of\;visit}$$

### 2.4. Statistics

Statistical analysis was performed using SPSS (v21.0; IBM, Armonk, NY, USA). Descriptive statistics for the participants’ characteristics were presented as mean (standard deviation, SD) for quantitative variables and frequency (percentage) for qualitative variables. The primary outcome of this study was the growth rate of body height (cm/day) across three stages—before, during and after the implementation of quarantine measures—calculated using five repeated measurements from the medical visit records of the study population. To compare quantitative variables across the different stages of the pandemic (pre-lockdown, during lockdown, and post-lockdown), a paired t-test was used. A p-value of less than 0.05 was considered statistically significant.

## 3. Results

A total of 72 study subjects were ultimately included in this research, consisting 26 males and 46 females. Descriptive statistics of the participants’ baseline characteristics are presented in Table 1. The average age of the participants was 9.75 years, with 73.6 % having a normal BMI. The average height of the fathers was 169.81 cm, while the mothers had an average height of 157.97 cm. The average birth weight was 2780.61 g, with an average gestational age of approximately 38.02 weeks. Among the participants, 76 % of the females and 62.6 % of the males were in the pubertal stage (Tanner stage >1.5).

**Fig. 2:**
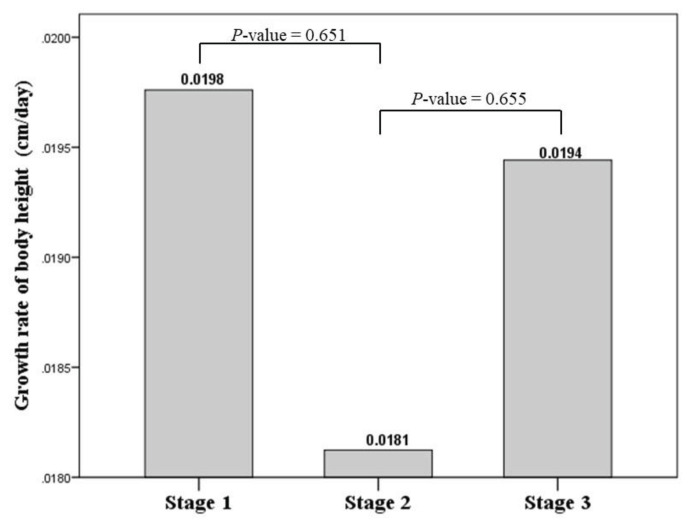
Mean of growth rate of body height in Stage 1 (pre-lockdown period), Stage 2 (during lockdown), and Stage 3 (post-lockdown period).

Fig. 2 illustrates the growth rates of children across different stages of the pandemic (pre-lockdown, during lockdown, and post-lockdown). Among the three stages, the lowest growth rate was observed during lockdown (stage 2) at 0.0181 cm/day, while the highest growth rate was observed prior to lockdown (stage 1), with a growth rate of 0.0198 cm/day. However, these differences were not statistically significant.

**Fig. 3:**
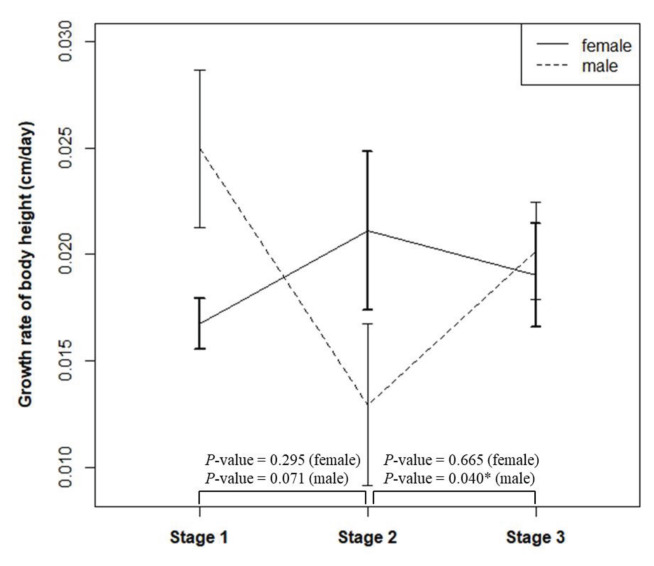
Comparison of growth rate differences between boys and girls in Stage 1 (pre-lockdown period), Stage 2 (during lockdown), and Stage 3 (post-lockdown period).

This study also investigated gender differences in growth rates across the three stages (pre-lockdown, during lockdown, and post-lockdown), as shown in Fig. 3. The results demonstrated a statistically significant difference in growth rates for males between the lockdown period (stage 2) and post-lockdown (stage 3), with a p-value of 0.04. Specifically, the growth rate for males increased after lockdown was lifted. In contrast, no statistically significant differences were observed in the growth rates for females across the three stages.

## 4. Discussion

This study found that PA positively contributes to linear growth in children and adolescents, particularly among males. Most research supports the beneficial effects of exercise on bone health; however, these findings often apply to specific subgroups (e.g., those with higher calcium intake, lower baseline activity levels, or smaller body size) [16]. Current studies examining the impact of PA on linear growth in children and adolescents primarily focus on select populations, such as athletes, or structured, skill-based activities like swimming, gymnastics, or dance. Differences across studies—in exercise intensity, duration, frequency, and age groups—and confounding factors, such as diet and sleep patterns, created challenges for conducting meta-analyses, making it difficult to draw definitive conclusions on the impact of PA on linear growth in children and adolescents [17,18]. Restrictions imposed during the COVID-19 pandemic provided a unique, natural opportunity to observe the impact of altered PA levels on linear growth. Several studies have since examined the negative effects of reduced PA on the growth of children and adolescents during the COVID-19 pandemic, often measuring height [19,20]. However, these studies lack consistency in measurement tools and recording methods, falling short of standardized, objective assessment protocols. For instance, some studies relied on growth assessments through questionnaires or telephone interviews, with height measurements conducted at home by parents, leading to a lack of professional, standardized, and objective assessments. In contrast, in this study, the linear growth of all participants was assessed by outpatient physicians at medical centers. Height measurements were conducted using stadiometers by trained doctors or nurses following standardized procedures [14], ensuring professional, consistent, and objective results. Compared to previous research, this methodology represents a significant distinction and advantage of our study. Possible mechanisms by which PA influences linear growth include its effects on the growth hormone (GH)-insulin-like growth factor-1 (IGF-1) axis, as well as endochondral ossification and growth plate senescence. PA stimulates GH secretion from the pituitary gland, directly impacting growth plate function and indirectly promoting IGF-1 production in the liver. GH also induces local IGF-1 production at the growth plate, which is more crucial for somatic growth than circulating IGF-1 from the liver [21]. During puberty, PA interacts with sex hormones, particularly estrogen, to enhance GH-driven chondrocyte proliferation, contributing to growth plate maturation and eventual closure [16,22]. Additionally, PA influences other hormones such as glucocorticoids; elevated levels of these hormones can reduce chondrocyte proliferation and downregulate GH and IGF-1 receptors [23]. Cytokines like IL-1β and IL-6 can also inhibit bone growth directly and by decreasing IGF-1 levels [4]. The effects of high-intensity exercise and the associated inflammatory response on child growth, especially in young athletes who exceed recommended activity levels, need further study to clarify long-term outcomes. Growth plates in children are particularly sensitive to both beneficial and adverse mechanical effects of PA. Chronic excessive PA or acute severe injury can damage growth plates, particularly between the ages of 10–16, potentially resulting in premature growth plate closure and permanent cessation of growth [24]. On the other hand, age-appropriate PA tailored to an individual’s developmental stage can protect growth plates and support overall health and growth [25]. Conversely, inadequate PA can negatively impact growth plates. Load-bearing tissues like cartilage may undergo atrophy without sufficient mechanical stimulation, indicating that a sedentary lifestyle could reduce growth potential, although the precise impact remains uncertain [26].

Our study indicated a significant deceleration in growth velocity among Taiwanese elementary school boys during the lockdown period, with statistically meaningful differences compared to preand post-lockdown phases. In contrast, growth velocity among elementary school girls showed no significant change across these three periods. This phenomenon may relate to inherent differences in PA levels between Taiwanese boys and girls. School closures and restrictions on public activity spaces during the pandemic impacted boys’ PA more profoundly, which likely contributed to the more noticeable effect on growth rates in boys compared to girls. Research highlights the direct influence of the school environment on children’s PA levels [27]. Children in school achieved an average daily moderate-to-vigorous PA ranging from 38.6 to 89.4 min, with similar findings for weekends. When using objective moderate-to-vigorous PA standards, variations in moderate-to-vigorous PA across schools accounted for 6 %–18 % of the daily variance [27]. Past research also suggested that the least active children tend to maintain lower activity levels over weekends [28]. Furthermore, sex-based differences in activity levels began to emerge in elementary school, with girls generally demonstrating lower PA levels than boys [29,30]. Longitudinal evidence also showed a more pronounced decline in girls’ PA during and after puberty, indicating that early sex differences may intensify through adolescence [31]. Other studies have found that boys aged 9 to 11 engage in more PA during recess than their female counterparts [32]. Ridgers et al. observed that boys spent approximately 30 % of recess time in MVPA, compared to only 24 % for girls [33]. Another study reported that boys engaged in MVPA during recess at a rate of 39.2 %, whereas girls did so at 23 % [34]. In Taiwan, research underscored a similar trend, with girls generally exhibiting lower PA levels than boys, emphasizing the need to further encourage PA among girls [35]. Additionally, younger children tend to participate in “chase” games (such as one child chasing another), while girls spend more time interacting with peers and/or teachers. Older boys, on the other hand, are more likely to engage in organized sports activities [36].

This study had several limitations. First, we did not directly measure PA levels before, during, and after the COVID-19 lockdown. Although we expected that students’ PA was restricted during the lockdown—since school closures forced them to remain at home—the actual degree of activity limitation may have varied among individuals due to differences in their home environments and lifestyles. Nonetheless, the COVID-19 restrictions provided a unique, natural opportunity to investigate the impact of altered physical activity on linear growth. Second, athletes were not excluded, which may have resulted in an underestimation of PA’s impact on linear growth. Previous studies suggest that athletes, unlike the general pediatric population, may exhibit catch-up growth during periods of reduced training time and intensity outside of competition or structured training [37]. Third, the study did not assess participants’ psychological state or sleep quality during the lockdown—factors likely affected by the pandemic and school closures, potentially inducing stress, anxiety, depression, or insomnia, all of which can influence linear growth [38,39]. This omission may have led to an overestimation of PA’s impact on linear growth. Prior research has highlighted increased psychological stress and sleep disturbances in children during lockdowns, and it is well-established that psychological stress and sleep quality can affect growth [38]. To minimize psychological influences on the study outcomes, this study excluded individuals with pre-existing psychological disorders or those newly diagnosed with conditions such as depression, autism, mood disorders, insomnia, anxiety or fear-related disorders, and post-traumatic stress disorder during the study period. Fourth, participants were not categorized into specific growth stages, such as prepubertal, early pubertal, pubertal growth spurt, or late pubertal phases. Growth rates vary significantly across these stages, particularly during the pubertal growth spurt [16], when biological and physiological factors like sex hormones may enhance linear growth, potentially masking the effects of PA. This limitation may have contributed to an underestimation of the true impact of PA on linear growth. Perhaps subsequent studies adding puberty and bone age [40] data to the integrated analysis can further confirm the contribution of PA to linear growth. Finally, this study has a small sample size and short follow-up intervals. The brief follow-up period at different stages may not adequately capture changes in growth rate, which, together with the limited number of participants, reduces the robustness of our conclusions. Future research with a larger sample and longer follow-up is necessary to validate these findings.

In conclusion, this study demonstrates that PA plays a crucial role in supporting linear growth in children, with boys experiencing a more marked impact. The lockdown-related reduction in PA notably affected growth rates, underscoring the value of maintaining active habits even under restrictive circumstances. These findings highlight the need to promote consistent physical activity for all children, with a particular focus on increasing engagement among girls to support their growth and overall health.

## Figures and Tables

**Table 1:** Baseline characteristics of demographic data.

	Subjects (N = 72)	Female (N = 46)	Male (N = 26)	P value
Gender				–
Female	46 (63.9 %)			
Male	26 (36.1 %)			–
Age	9.75 (2.34)	9.41 (1.75)	10.35 (3.07)	0.163
Body Height	136.91 (13.28)	135.65 (11.12)	139.14 (16.44)	0.340
Body Weight	33.40 (9.81)	32.33 (9.37)	35.29 (10.47)	0.222
Body Mass Index	17.42 (3.13)	17.21 (2.88)	17.80 (3.54)	0.442
Normal	53 (73.6 %)	34 (73.9 %)	19 (73.1 %)	0.286
Overweight	10 (13.9 %)	8 (17.4 %)	2 (7.7 %)	
Obesity	5 (6.9 %)	3 (6.5 %)	2 (7.7 %)	
Underweight	4 (5.6 %)	1 (2.2 %)	3 (11.5 %)	
Parents’ height (mother)	157.97 (6.09)	158.77 (5.79)	156.44 (6.48)	0.137
Parents’ height (father)	169.81 (6.27)	170.71 (6.74)	168.09 (4.94)	0.105
Body weight of birth (g)	2780.61 (615.18)	2746.48 (697.95)	2845.62 (422.66)	0.554
Weeks of gestation	38.02 (3.28)	37.68 (3.91)	38.65 (1.54)	0.229
Tanner stage (Breast)				0.235
1	8 (11.1 %)	8 (17.8 %)	0 (0 %)	
1.5	3 (4.2 %)	3 (6.7 %)	0 (0 %)	
2	22 (47.8 %)	22 (48.9 %)	0 (0 %)	
2.5	6 (13.0 %)	5 (11.1 %)	1 (100 %)	
3	6 (13.0 %)	6 (13.3 %)	0 (0 %)	
4	1 (2.2 %)	1 (2.2 %)	0 (0 %)	
Tanner stage (Genitalia)				
1	8 (33.3 %)	–	8 (33.3 %)	
1.5	1 (4.2 %)	–	1 (4.2 %)	
2	3 (12.5 %)	–	3 (12.5 %)	
2.5	1 (4.2 %)	–	1 (4.2 %)	
3	8 (33.3 %)	–	8 (33.3 %)	
3.5	1 (4.2 %)	–	1 (4.2 %)	
4	1 (4.2 %)	–	1 (4.2 %)	
4.5	1 (4.2 %)	–	1 (4.2 %)	
Tanner stage (Pubic hair)				
1	53 (76.8 %)	40 (88.9 %)	13 (54.2 %)	0.009
2	9 (13.0 %)	3 (6.7 %)	6 (25.0 %)	
2.5	1 (1.4 %)	1 (2.2 %)	0 (0 %)	
3	4 (5.8 %)	1 (2.2 %)	3 (12.5 %)	
4	2 (2.9 %)	0 (0 %)	2 (8.3 %)	
Tanner stage (Pubic hair)				
1	53 (76.8 %)	40 (88.9 %)	13 (54.2 %)	0.002
>1		5 (11.1 %)	11 (45.8 %)	
Bone age (months)	58.84 (60.68)	53.13 (57.22)	67.94 (66.59)	0.437

Data presented as N (%) or mean (SD).

## References

[1] World Health Organization Training course on child growth assessment Geneva, Switzerland World Health Organization 2008

[2] Alves JGB Alves GV Effects of physical activity on children’s growth J Pediatr (Rio J) 2019 95 Suppl 1 72 8 10.1016/j.jped.2018.11.003 30593790

[3] Jazbinšek S Kotnik P Influence of physical activity on linear growth in children and adolescents Ann Kinesiol 2020 11 129 10.35469/ak.2020.222

[4] Jee YH Baron J The biology of stature J Pediatr 2016 173 32 8 10.1016/j.jpeds.2016.02.068 27025909 PMC4884478

[5] Chahar PS Physiological basis of growth and development among children and adolescents in relation to physical activity Am J Sports Sci Med 2014 2 17 22 10.12691/ajssm-2-5A-5

[6] Silva CC Goldberg TBL Teixeira AS Marques I Does physical exercise increase or compromise children’s and adolescents’ linear growth? Is it a myth or truth? Rev Bras Med Esporte 2004 10 525 8

[7] Caspersen CJ Powell KE Christenson GM Physical activity, exercise, and physical fitness: definitions and distinctions for health-related research Publ Health Rep 1985 100 126 31 PMC1424733 3920711

[8] Poitras VJ Gray CE Borghese MM Carson V Chaput JP Janssen I Systematic review of the relationships between objectively measured physical activity and health indicators in school-aged children and youth Appl Physiol Nutr Metab 2016 41 S197 239 10.1139/apnm-2015-0663 27306431

[9] World Health Organization Global recommendations on physical activity for health Geneva, Switzerland World Health Organization 2010 26180873

[10] Bielemann RM Martinez-Mesa J Gigante DP Physical activity during life course and bone mass: a systematic review of methods and findings from cohort studies with young adults BMC Muscoskelet Disord 2013 14 77 10.1186/1471-2474-14-77 PMC3599107 23497066

[11] Frois RR de S Pereira LA Cardeal CM Asano RY Bartholomeu Neto J Oliveira JF Resistance training for children: a meta-analysis of longitudinal changes in growth, strength and body composition Rev Bras Ciência Mov 2014 22 137 49 [In Spanish, English abstract]

[12] Neville RD Lakes KD Hopkins WG Tarantino G Draper CE Beck R Global changes in child and adolescent physical activity during the COVID-19 pandemic: a systematic review and meta-analysis JAMA Pediatr 2022 176 886 94 10.1001/jamapediatrics.2022.2313 35816330 PMC9274449

[13] Rossi L Behme N Breuer C Physical activity of children and adolescents during the COVID-19 Pandemic-A scoping review Int J Environ Res Publ Health 2021 18 11440 10.3390/ijerph182111440 PMC8583307 34769956

[14] Foote JM Optimizing linear growth measurement in children J Pediatr Health Care 2014 28 413 9 10.1016/j.pedhc.2014.01.001 24560628

[15] Irwin M Lazarevic B Soled D Adesman A The COVID-19 pandemic and its potential enduring impact on children Curr Opin Pediatr 2022 34 107 15 10.1097/MOP.0000000000001097 34923563 PMC8728751

[16] Slemenda CW Reister TK Hui SL Miller JZ Christian JC Johnston CC Jr Influences on skeletal mineralization in children and adolescents: evidence for varying effects of sexual maturation and physical activity J Pediatr 1994 125 201 7 10.1016/s0022-3476(94)70193-8 8040762

[17] Rutten C Boen F Seghers J Which school- and home-based factors in elementary school-age children predict physical activity and sedentary behavior in secondary school-age children? A prospective cohort study J Phys Activ Health 2015 12 409 17 10.1123/jpah.2013-0128 24770355

[18] Bass S Bradney M Pearce G Hendrich E Inge K Stuckey S Short stature and delayed puberty in gymnasts: influence of selection bias on leg length and the duration of training on trunk length J Pediatr 2000 136 149 55 10.1016/s0022-3476(00)70094-1 10657818

[19] Koletzko B Holzapfel C Schneider U Hauner H Lifestyle and body weight consequences of the COVID-19 pandemic in children: increasing disparity Ann Nutr Metab 2021 77 1 3 10.1159/000514186 PMC7900479 33498055

[20] Han JA Chung YE Chung IH Hong YH Chung S Impact of the COVID-19 pandemic on seasonal variations in childhood and adolescent growth: experience of pediatric Endocrine Clinic Child (Basel) 2021 8 404 10.3390/children8050404 PMC8155986 34067734

[21] Domené HM Martínez AS Frystyk J Bengolea SV Ropelato MG Scaglia PA Normal growth spurt and final height despite low levels of all forms of circulating insulinlike growth factor-I in a patient with acid-labile subunit deficiency Horm Res 2007 67 243 9 10.1159/00009847 17213728

[22] Weise M De-Levi S Barnes KM Gafni RI Abad V Baron J Effects of estrogen on growth plate senescence and epiphyseal fusion Proc Natl Acad Sci U S A 2001 98 6871 6 10.1073/pnas.121180498 11381135 PMC34445

[23] Riddell MC The endocrine response and substrate utilization during exercise in children and adolescents J Appl Physiol (1985) 2008 105 725 33 10.1152/japplphysiol.00031.2008 18420724

[24] Laor T Wall EJ Vu LP Physeal widening in the knee due to stress injury in child athletes AJR Am J Roentgenol 2006 186 1260 4 10.2214/AJR.04.1606 16632716

[25] Carson BP The potential role of contraction-induced myokines in the regulation of metabolic function for the prevention and treatment of type 2 diabetes Front Endocrinol 2017 8 97 10.3389/fendo.2017.00097 PMC5411437 28512448

[26] Mirtz TA Chandler JP Eyers CM The effects of physical activity on the epiphyseal growth plates: a review of the literature on normal physiology and clinical implications J Clin Med Res 2011 3 1 7 10.4021/jocmr477w 22043265 PMC3194019

[27] Salway R Emm-Collison L Sebire SJ Thompson JL Lawlor DA Jago R A multilevel analysis of neighbourhood, school, friend and individual-level variation in primary school children’s physical activity Int J Environ Res Publ Health 2019 16 4889 10.3390/ijerph16244889 PMC6950546 31817182

[28] Jago R Salway R Lawlor DA Emm-Collison L Heron J Thompson JL Profiles of children’s physical activity and sedentary behaviour between age 6 and 9: a latent profile and transition analysis Int J Behav Nutr Phys Act 2018 15 103 10.1186/s12966-018-0735-8 30352597 PMC6199754

[29] Forthofer M Dowda M O’Neill JR Addy CL McDonald S Reid L Effect of child gender and psychosocial factors on physical activity from fifth to sixth grade J Phys Activ Health 2017 14 953 8 10.1123/jpah.2016-0487 28682693

[30] Trost SG Pate RR Sallis JF Freedson PS Taylor WC Dowda M Age and gender differences in objectively measured physical activity in youth Med Sci Sports Exerc 2002 34 350 5 10.1097/00005768-200202000-00025 11828247

[31] Vilhjalmsson R Kristjansdottir G Gender differences in physical activity in older children and adolescents: the central role of organized sport Soc Sci Med 2003 56 363 74 10.1016/s0277-9536(02)00042-4 12473321

[32] Owen CG Nightingale CM Rudnicka AR Cook DG Ekelund U Whincup PH Ethnic and gender differences in physical activity levels among 9–10-year-old children of white European, South Asian and African-Caribbean origin: the Child Heart Health Study in England (CHASE Study) Int J Epidemiol 2009 38 1082 93 10.1093/ije/dyp176.33 19377098 PMC2720395

[33] Ridgers ND Salmon J Parrish AM Stanley RM Okely AD Physical activity during school recess: a systematic review Am J Prev Med 2012 43 320 8 10.1016/j.amepre.2012.05.019 22898126

[34] Ridgers ND Stratton G Fairclough SJ Assessing physical activity during recess using accelerometry Prev Med 2005 41 102 7 10.1016/j.ypmed.2004.10.023 15917000

[35] Chen MY Chou CC Yang RJ Considering the factors of gender and body weight in the promotion of healthy behavior among adolescents J Nurs Res 2005 13 235 43 10.1097/01.jnr.0000387545.76007.8b 16237635

[36] Shervey SW DiPerna JC Engagement in physical activity during recess: gender and grade level differences in the elementary grades J Phys Activ Health 2017 14 677 83 10.1123/jpah.2014-0499 28513317

[37] Roemmich JN Rogol AD Physiology of growth and development. Its relationship to performance in the young athlete Clin Sports Med 1995 14 483 502 7553919

[38] Araújo LA Veloso CF Souza MC Azevedo JMC Tarro G The potential impact of the COVID-19 pandemic on child growth and development: a systematic review J Pediatr (Rio J) 2021 97 369 77 10.1016/j.jped.2020.08.008 32980318 PMC7510529

[39] Viner R Russell S Saulle R Croker H Stansfield C Packer J School closures during social lockdown and mental health, health behaviors, and well-being among children and adolescents during the first COVID-19 wave: a systematic review JAMA Pediatr 2022 176 400 9 10.1001/jamapediatrics.2021.584 35040870

[40] Cheng CF Huang ET Kuo JT Liao KY Tsai FJ Report of clinical bone age assessment using deep learning for an Asian population in Taiwan Biomedicine (Taipei) 2021 11 50 8 10.37796/2211-8039.1256 35223411 PMC8823497

